# Effect of olive oil administration on the level of transforming growth factor β1 during orthodontic tooth movement in old and young guinea pigs

**DOI:** 10.12688/f1000research.21177.2

**Published:** 2020-05-07

**Authors:** Sri Suparwitri, Paramita Noviasari

**Affiliations:** 1 Department of Orthodontics, Faculty of Dentistry, Universitas Gadjah Mada, Yogyakarta, 55281, Indonesia

**Keywords:** olive oil, transforming growth factor β, orthodontic tooth movement

## Abstract

**Background:** Orthodontic tooth movement occurs due to continuous pressure on the teeth, causing the remodeling of the alveolar bone. The tissue will respond to bone growth factors, such as transforming growth factor β1 (TGF-β1), fibroblasts, and bone morphogenetic proteins, for new bone formation. The remodeling process is balanced in young adults, but there is an imbalance in older individuals due to decreased differentiation, activity, and life span of osteoblasts, and increased osteoclasts. Olive oil contains lots of antioxidants and can slow down the aging process. This study aims to study the differences in TGF-β1 levels between old and young guinea pigs, and the difference between olive oil administration on TGF-β1 levels in old and young guinea pigs with orthodontic tooth movement.

**Methods:** 12 guinea pigs divided into 4 groups: young guinea pigs (4-5 months) not given olive oil; young guinea pigs given olive oil; old guinea pigs (30-31 months) given olive oil; old guinea pigs given olive oil. The teeth were moved using an open coil spring mounted on the brackets on both lower incisors. Gingival sulcus fluid samples were taken on days 0, 7 and 14 of the movement of the teeth. TGF-β1 levels were analyzed using ELISA.

**Results: **Three-way ANOVA and post hoc statistical tests showed that TGF-β1 levels in young guinea pigs were significantly higher than old guinea pigs on days 0, 7 and 14 (p<0.05). TGF-β1 levels in both young and old guinea pigs who were given olive oil was significantly higher than those not given olive oil on days 0, 7 and 14 (p<0.05).

**Conclusions**: TGF-β1 levels in the younger age guinea pigs were higher than the older age, and olive oil could increase TGF-β1 levels in the older age guinea pigs.

## Introduction

Orthodontic treatment aims to correct malposition, malocclusion, and malrelation of dentoskeletal abnormality to get harmony, balance in aesthetics, face and head structure. Orthodontic tooth movement is a result of the remodeling of periodontal ligaments and alveolar bone ^1^. Bone remodeling during orthodontic tooth movement is described as a continuous and balanced process ^2^. The process of bone remodeling is regulated by osteoblasts and osteoclasts ^3^. Osteoclasts in pressure areas will resorb the alveolar bone so that tooth movement occurs, and this phenomenon will be balanced by osteoblasts in the tension area ^4^. Transforming growth factor-β1 (TGF-β1) is known as a multifunctional cytokine that plays an important role in bone formation. Moreover, TGF-β1 activity increases with bone formation ^5^.

The need for orthodontic treatment in adulthood increases with age, but the balance of bone resorption and remodeling processes decreases with increasing age ^6^. Therefore, cellular activity in periodontal tissues of young and old individuals needs to be further studied. This may greatly influence the success of orthodontic treatment ^7^. The process of aging is caused by free radicals, hormones, genetics, lifestyle, diet, pollution, and socioeconomic conditions. If these causative factors can be avoided, then the aging process can be prevented, slowed down, and may even be inhibited ^8^.

Food ingredients that contain antioxidants can inhibit the oxidation process in the aging process. The oxidation process can increase intracellular reactive oxygen activity so that it can inhibit bone remodeling ^9^. Olive oil is a food ingredient that rich of antioxidants and is often consumed by the public. Olive oil which extracted from fresh olives have been shown to slow the aging process ^10^. According to Liu *et al.,* olive oil acts effectively as a substitute for estrogen to prevent bone loss ^11^. Melguizo-Rodríguez *et al.*, proved the phenolic compound in virgin olive oil extracts can increase the proliferation and differentiation of osteoblasts. Phenolic compounds (caffeic acid, ferulic acid, coumaric acid, apigenin, and luteolin) in olive oil extracts can increase TGF-β1 and its receptor ^12^.

Therefore, olive oil is expected to reduce osteoclastogenesis so as to reduce tooth movement orthodontics, especially for cases to prevent loss of anchorage, periodontitis and tooth retention. The purpose of this study is to study the differences in TGF-β1 levels between old and young ages, using guinea pig models, and the effect of olive oil administration on TGF-β1 levels in old and young guinea pigs with orthodontic tooth movement. The guinea pig was chosen as an animal model as it has been previously used to study the effect of medications on orthodontic tooth movement and has the potential to simulate the response of human tissue ^13^.

## Methods

### Ethical clearance

Ethical clearance was approved by the Research Ethics Committee of the Faculty of Dentistry, University Gadjah Mada (UGM), Yogyakarta, Indonesia (00144/KKEP/FKG-UGM/EC/2019). “Animal Research: Reporting of *in vivo* Experiments” (ARRIVE) was used for reporting this study. During orthodontic appliance installation and gingival crevicular fluid collection, all the animals were anesthetized. We also used non-invasive techniques to move the teeth (using minimal 35 gr force, which is acceptable for guinea pig), and housing conditions that provided a comfortable and safe environment. All these procedures were done to ameliorate any suffering of animals used in this study.

### Animals

We used 12 male guinea pigs ( *Cavia porcellus*) (purchased from a commercial supplier, Kaliurang, Yogyakarta, Indonesia), which were chosen utilizing simple random sampling into four groups (n=3/group): young guinea pigs that were not administered olive oil (young guinea pig-control); young guinea pigs that were administered olive oil; old guinea pigs that were not administered olive oil (old guinea pig-control); old guinea pigs administered olive oil. Young guinea pigs were 4–5 months old and weighed approximately 300–400 grams, whilst old guinea pigs were 30–31 months old and weighed approximately 800–1000 grams. Sample size for the groups (n=3) was obtained utilizing Lemeshow's formula ^14^. A sample size of three animals in each group would give more than 80% power to detect significant differences with 0.45 effect size and at a significance level of α = 0.05. Sample groups were selected by means of simple random number sampling. Each animal was assigned a tag number, the blind-folded researcher then picks numbered tags from the hat.

All the animals were maintained for a week before the experiment began on an individual polycarbonate cage (30 cm × 20 cm × 20 cm) with a 12-h light/dark cycle at a steady temperature of 25°C and humidity of 50% for acclimatization to compensate for their various origins. All animals were fed a standard pellet diet (expanded pellets; Stepfield, UK) with tap water *ad libitum*.

### Calculation of olive oil dosage and administration

**Figure 1.**
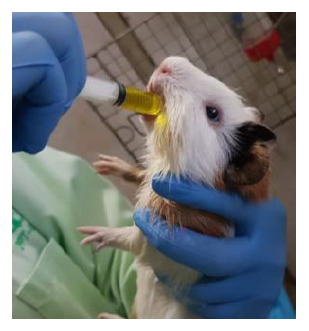
Administration of olive oil to a guinea pig.

We used extra virgin olive oil (Bertolli®, USA). Olive oil consumed by humans per day is 30–50 grams so the conversion of a human dose (70 kg) to guinea pig (400 grams) is 0.031. The dose was converted from human olive oil dose (weight, 70 kg) to a guinea pig dose (weight, 400g): 0.031 × 30 ml = 0.93 ml ^15^. Based on body weight, the dose of olive oil given to young guinea pigs (300–400 g) was 0.7 ml and for old guinea pigs (800–1000 g) was 1.86 ml. Olive oil treatment groups (see below) were given olive oil daily from the beginning of the installation of orthodontic devices until day 14, while control groups received mock oil. Administration of oil was carried out orally with injection syringes that have been cut off ( Figure 1) and were given at 9am every morning.

### Orthodontic installation

**Figure 2.**
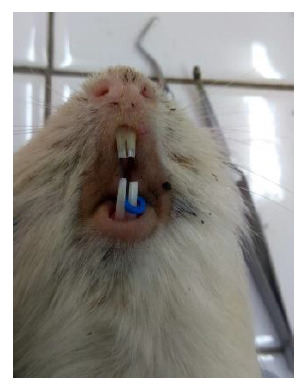
Separator installation on a guinea pig to facilitate the installation of the bracket.

**Figure 3.**
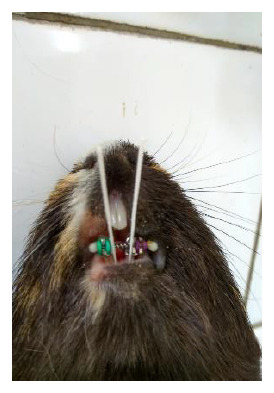
Installation of bracket and gingival crevicular fluid isolation.

Guinea pigs were first anesthetized with ketamine (160095, Kepro™, Netherlands; 35mg/kgBW) and xylazine (160096, Xyla™, Netherlands; 5mg/kgBW) intramuscularly on the thigh, then a separator was placed between the two lower incisors ( Figure 2). Next, the teeth were cleaned with rubber cups and pumice (22257, Kerr, USA) and an incisivus Roth bracket 0.022” (American Orthodontics, USA) was bonded on both lower guinea pig’s incisor using an orthodontic adhesive (Transbond XT, 3M Unitek, USA), followed by light-curing for 40 s according to the manufacturer (10 s for each side: mesial, occlusal, gingival, and distal) using a quartz-tungsten-halogen (QTH) light-curing unit (Litex 680A, Dentamerica, USA) with a light intensity of 450 mW/cm ^2^. The wire arch mounted was 0.018” stainless steel wire arch, which was ligated using power O (American orthodontics, USA). Open coil spring NiTi (American Orthodontics, USA) was installed between the two brackets to move the teeth distally with a force of 0.35 N ( Figure 3). The magnitude of the open coil spring force was measured using a tension gauge. The length of the stainless-steel wire was cut 4 mm longer than the open coil spring as a tolerance for orthodontic tooth movement.

### Isolation of the gingival crevicular fluid

Gingival crevicular fluid (GCF) collection was performed on days 0, 7, and 14 after olive oil administration. Before removing GCF, the lower incisors of the guinea pig were cleaned with a cotton swab to remove supragingival plaque. Then the teeth were isolated with cotton wool and dried to exclude the remaining saliva. Next, a #15 paper point (Paper Points ISO 0.2, Dentsply, Germany) was inserted into the gingival sulcus of the mandibular incisor about 1 mm deep for 30 seconds to absorb all the GCF fluid and repeated thrice at an interval of 90 seconds to increase the volume of GCF fluid isolated ( Figure 3). The paper point was then inserted into a 1.5 ml eppendorf tube containing 350 μl of saline solution. The eppendorf tube was centrifuged for 5 minutes at a speed of 2000 rpm to elucidate complete GCF components. Paper points were removed and the supernatant solution was stored at -80°C until required for analysis.

### Detection TGF-β1 levels

Detection of TGF-β1 levels was carried out at the Molecular Biology Laboratory of the Faculty of Medicine UGM using ELISA (enzyme-linked immunosorbent assay). The GCF was analyzed for TGF-β1 levels using ELISA kit reagent test (Cusabio, China), according to manufacturer protocols.

### Data analysis

Three-way analysis of variance followed by post hoc statistical tests was used to analyse the difference among the groups and determine its significance. *P*-values <0.05 were considered statistically significant. All of the data were analyzed using SPSS software (Statistical Package for the Social Sciences, v22.0, USA).

## Results

All the experimental procedures were well-tolerated. Furthermore, olive oil administration at the used dosage did not induce any general toxicity, including edema. The results of measurements of TGF-β1 levels of GCF before and after tooth movement can be seen in Table 1. The mean TGF-β1 levels in the older age groups on days 0, 7 and 14 were higher than in the younger age groups, both in the olive oil groups and control groups. The mean TGF-β1 levels in the older and younger age groups increased on day 7 and decreased on day 14 ( Table 1).

**Table 1.** TGF-β1 levels in gingival crevicular fluid among the two groups tested.

	Age	Group	Day 0	Day 7	Day 14
**TGF-β1 (pg/ml)**	Old	Control	23.19 ± 4.610	28.47 ± 4.648	23.51 ± 3.283
		*Olive Oil*	37.75 ± 4.052	49.92 ± 1.469	45.57 ± 3.330
	Young	Control	39.57± 3.754	53.84 ± 3.775	50.46 ± 4.732
		*Olive Oil*	50.56± 2.570	75.32 ± 1.515	66.14 ± 3.349

**Figure 4.**
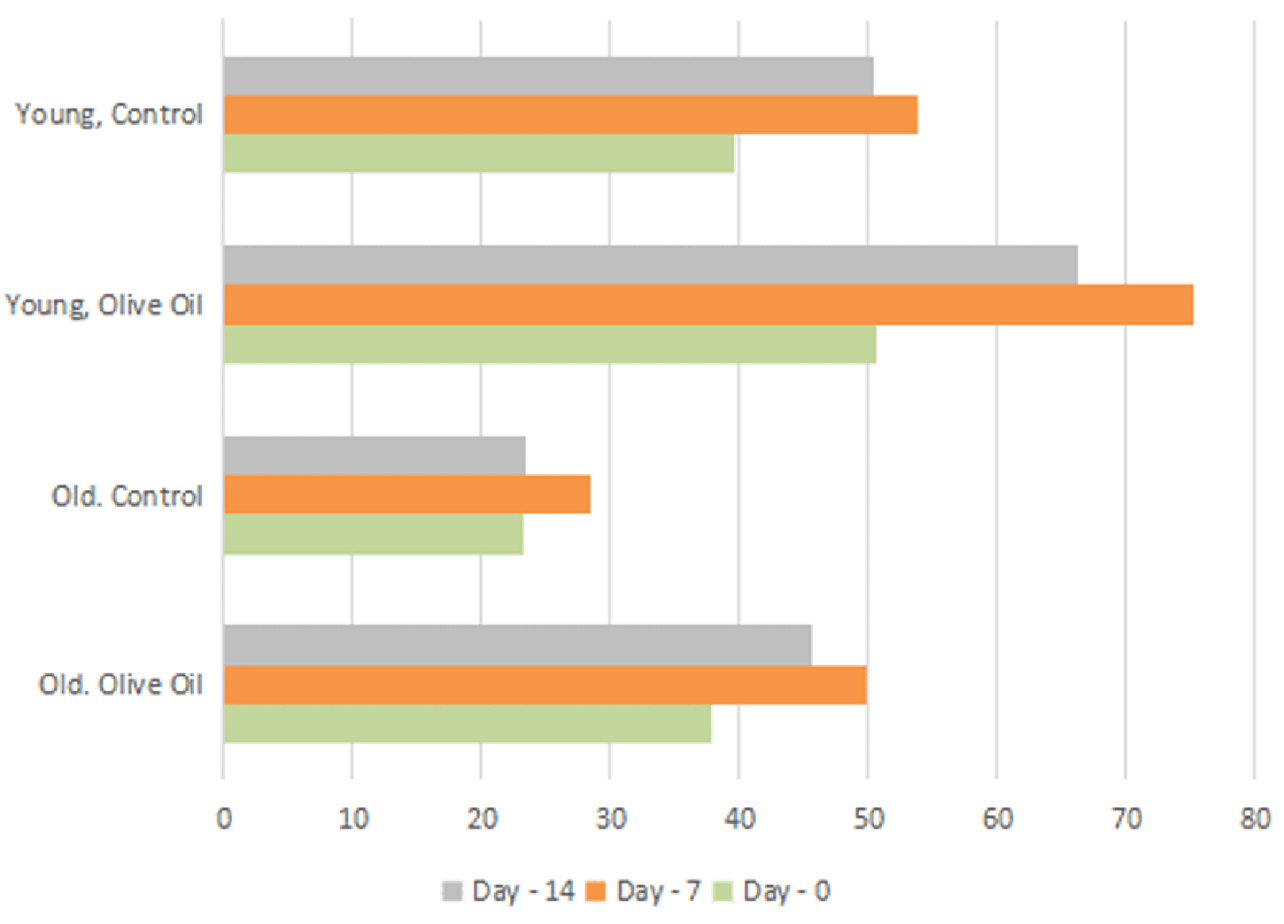
TGF-β1 levels (pg/ml) in gingival crevicular fluid of guinea pigs with tooth movement treated and untreated with olive oil.

Figure 4 shows the administration of olive oil increases the average level of TGF-β1 in the young and old age groups. The mean TGF-β1 levels in the olive oil group were higher than the mean TGF-β1 in the group that were not given olive oil (control). The mean TGF-β1 levels in the old control and treatment groups and were higher than the young control and treatment on all days. Based on the time of observation, the highest TGF-β1 level was seen on day 7 and the lowest on day 0 in all groups. The highest TGF-β1 level was found in the young guinea pig group treated with olive oil at day 7 and the lowest TGF-β1 level was found in the old guinea pig group who were not given olive oil (young control) on day 0.

Normality test (Saphiro-Wilk) and homogeneity test (Levene) showed normally distributed and homogeneous data. The research data were then analyzed using three-way ANOVA (Analysis of variance). The results of the three-way ANOVA ( Table 2) showed that age, olive oil administration and observation time had a significant effect on TGF-β1 levels *(p* <0.05). There was an interaction between administration of olive oil and observation time, and also between observation time and age, but there was no interaction between administration of olive oil and age or administration of olive oil, observation time, and age ( *p*> 0.05).

**Table 2.** Results of three-way ANOVA of TGF-β1 secretion in gingival crevicular fluid of old and young guinea pigs with teeth movement who were given olive oil and controls.

Variables	Significance
*Olive oil*	0.000*
Days of observation	0.000*
Age	0.000*
*Olive oil*- Days of observation	0.018*
*Olive oil* – Age	0.140
Days of observation – Age	0.002*
*Olive oil* - Days of observation – Age	0.461

A post hoc test was performed after ANOVA to see groups that had significant differences (p <0.05). The post hoc test results showed that most of the TGF-β1 activity of the old and young age groups who were given olive oil and not given olive oil on days 0, 7, and 14 showed significant differences ( Table 3).

**Table 3.** Post hoc test results of mean TGF-β1 secretion in gingival crevicular fluid in old and young guinea pigs given olive oil and controls.

	OO D-0	OO D-7	OO D-14	OC D-0	OC D-7	OC D-14	YO D-0	Y0 D-7	YO D-14	YC D-0	YC D-7	YC D-14
OO D-0	-											
OO D-7	0.000 *	-										
OO D-14	0.013 *	0.150	-								
OC D-0	0.000 *	0.000 *	0.000 *	-								
OC D-7	0.004 *	0.000 *	0.000	0.084	-							
OC D-14	0.000 *	0.000 *	0.000 *	0.819	0.053	-						
YO D-0	0.000 *	0.830	0.102	0.000 *	0.000 *	0.000 *	-					
YO D-7	0.000 *	0.000 *	0.000 *	0.000 *	0.000 *	0.000 *	0.000 *	-				
YO D14	0.000 *	0.000 *	0.000 *	0.000 *	0.000 *	0.000 *	0.000 *	0.004 *	-			
YC D-0	0.540	0.002 *	0.051	0.000 *	0.001 *	0.000 *	0.001 *	0.000 *	0.000 *	-		
YC D-7	0.000 *	0.193	0.009 *	0.000 *	0.000 *	0.000 *	0.273	0.000 *	0.000 *	0.000 *	-	
YC D-14	0.000 *	0.856	0.108	0.000 *	0.000 *	0.000 *	0.974	0.000 *	0.000 *	0.001 *	0.259	-

* = significantly different p<0.05; OC = old, control; OO = Old, olive oil; YC = Young, control; YO = Young, olive oil.

## Discussion

The process of bone remodeling is regulated by osteoblasts and osteoclasts. Osteoblasts and osteoclasts will appear 40–48 hours after being exposed with orthodontic forces ^16^. Osteoclasts in depressed areas will absorb alveolar bone so that tooth movement occurs. This phenomenon will be offset by the apposition of osteoblasts in the tension area ^2^. The balance of bone resorption and remodeling processes decreases with increasing age, which greatly influences the success of orthodontic treatment ^6, 7^. Olive oil has been shown to contains antioxidant compounds, namely phenolic compounds. Phenolic compounds are able to provide a strong protective effect against aging ^17– 19^. The results of the current data analysis showed that the average TGF-β1 level in GCF in the olive oil groups was higher than the mean TGF-β1 in the groups that was not given olive oil (controls). The results of the three-way ANOVA ( Table 2) showed that age, olive oil administration and observation time had a significant effect on TGF-β1 levels ( *p*<0.05). TGF-β1 is a multifunctional cytokine that modulates proliferation, growth, differentiation, adhesion and cell survival, but it also plays a role in the production of extracellular matrix proteins ^5^. The application of orthodontic forces can activate TGF-β1 transcription in these regions, thereby increasing TGF-β1 levels in GCF ^12^. TGF-β1 can recruit osteoblasts precursors in the area of bone formation and stimulate osteoblasts to differentiate to produce bone matrix ^20^. During tooth movement, latent TGF-β1 is activated in the periodontal tissue by plasmin, which is produced by retracted periodontal ligament cells and set in the cascade of plasminogen activator ^5^. During bone formation, TGF-β1 inhibits the recruitment of osteoclast precursor cells and directly inhibits osteoclast activity in bone resorption ^18^. Moreover, TGF-β1 in this area will stimulate osteoblastogenesis and new bone formation together with inhibition of osteoclastogenesis in this area ^5, 19^. Previous study showed that TGF-β1 can stimulate osteoclast apoptosis in the area. The effect of TGF β proapoptosis is mediated by several factors such as osteoblasts and stromal cells under TGF-β1 control ^5^.

The phenolic content in virgin olive oil extracts such as apigenin, luteolin, coumaric acid, ferulic acid and affeic acid can increase the proliferation capacity and differentiation of osteoblasts ^12^. Inhibition of osteoclastogenesis is also seen in bone resorption markers, such as OPG(osteoprotegerin) and Receptor Activator of Nuclear Factor-KappaB Ligand (RANKL) ^2^. The phenolic content in virgin olive oil extract can improve bone repair, and this is related to osteoblastogenesis and related factors, such as transcription factor 2 (RUNX-2), osterix (OSX), collagentype I (COL I) osteocalcin (OSC), and alkalinephosphatase (ALP). The phenolic compounds contained in virgin olive oil extract significantly increase the levels of TGFβ-1 and its receptors, and TGFβ-1 receptor expression is significantly increased in the presence of phenolic compounds ^12^.

Tooth movement in orthodontic treatment can be divided into three stages: initial phase, lag phase, and post lag phase. In the initial phase, the orthodontic force will produce 0.4-0.9 mm of tooth movement. This phase takes place within one week. Cellular reaction starts after the application of force, this is seen by the presence of osteoclasts, osteoblasts, osteoblast progenitors and inflammatory cells ^16^. In the present study, TGFβ-1 levels of in the young and old guinea pigs reached the maximum value on observation day 7 indicating that the movement of the teeth is in the initial phase. Decreased levels of TGFβ-1 on day 14 indicate that tooth movement is in the lag phase. The lag phase is characterized by no or minimal movement of the teeth ^21^.

## Conclusion

Olive oil increases TGFβ-1 levels in both young and old guinea pigs during orthodontic tooth movement. Further studies are necessary at clinical levels to confirm the efficacy and potency of olive oil in increasing bone remodeling in orthodontic patients, especially in older patients.

## Data availability

Figshare: Picture1.jpg, https://doi.org/10.6084/m9.figshare.10095101 ^22^

This project contains the following underlying data:

- Raw data.xlsx (raw data of TGF beta levels among two groups tested)
- Statistic TGF beta.docs (statistic data)
- Fig 1 (raw image used for Figure 1)
- Fig 2 (raw image used for Figure 2)
- Fig 3 (raw image used for Figure 3)

Data are available under the terms of the Creative Commons Attribution 4.0 International license (CC-BY 4.0).
