# Supplementary material for: Effect of olive oil administration on the level of transforming growth factor β1 during orthodontic tooth movement in old and young guinea pigs
Source: F1000Res. 2020 May 7;8:2028. Originally published 2019 Nov 29. [Version 2] doi: 10.12688/f1000research.21177.2 (PMC7295082; doi:10.12688/f1000research.21177.2)
Supplement: Peer review history

# Peer review: Reviewer response for version 2

**DOI:** 10.5256/f1000research.26325.r63180

**Published:** 2020-06-12

**Authors:** Hong He, Mingyuan Du

**Affiliations:** 1 The State Key Laboratory Breeding Base of Basic Science of Stomatology (Hubei‐MOST) & Key Laboratory of Oral Biomedicine Ministry of Education, School & Hospital of Stomatology, Wuhan University, Wuhan, China

Our initial questions were well addressed in the revised version, and we have no further comments to make.

Is the work clearly and accurately presented and does it cite the current literature?

Partly

If applicable, is the statistical analysis and its interpretation appropriate?

I cannot comment. A qualified statistician is required.

Are all the source data underlying the results available to ensure full reproducibility?

Yes

Is the study design appropriate and is the work technically sound?

Partly

Are the conclusions drawn adequately supported by the results?

Yes

Are sufficient details of methods and analysis provided to allow replication by others?

Partly

Reviewer Expertise:

Mechanisms of orthodontic tooth movement

We confirm that we have read this submission and believe that we have an appropriate level of expertise to confirm that it is of an acceptable scientific standard.

# Peer review: Reviewer response for version 2

**DOI:** 10.5256/f1000research.26325.r63181

**Published:** 2020-05-11

**Authors:** Ervina Sofyanti, Denny Satria

**Affiliations:** 1 Department of Pharmaceutical Biology, Faculty of Pharmacy, Universitas Sumatera Utara, Medan, Indonesia; 2 Department of Orthodontics, Faculty of Dentistry, Universitas Sumatera Utara , Medan, Indonesia

We have no further comments to make. 

 Thank you.

Is the work clearly and accurately presented and does it cite the current literature?

Partly

If applicable, is the statistical analysis and its interpretation appropriate?

Yes

Are all the source data underlying the results available to ensure full reproducibility?

Partly

Is the study design appropriate and is the work technically sound?

Yes

Are the conclusions drawn adequately supported by the results?

Partly

Are sufficient details of methods and analysis provided to allow replication by others?

Partly

Reviewer Expertise:

Ervina Sofyanti :  The findings that can influence the perception of urgency of integrated medical orthodontics approach; Denny Satria : molecular and cell biology in clinical cases that might require multidisciplinary study.

We confirm that we have read this submission and believe that we have an appropriate level of expertise to confirm that it is of an acceptable scientific standard.

# Peer review: Reviewer response for version 1

**DOI:** 10.5256/f1000research.23310.r60725

**Published:** 2020-03-09

**Authors:** Hong He, Mingyuan Du

**Affiliations:** 1 The State Key Laboratory Breeding Base of Basic Science of Stomatology (Hubei‐MOST) & Key Laboratory of Oral Biomedicine Ministry of Education, School & Hospital of Stomatology, Wuhan University, Wuhan, China

The paper titled "Effect of olive oil administration on the level of transforming growth factor β1 during orthodontic tooth movement in old and young guinea pigs " showed that TGF-β1 levels in the younger age guinea pigs were higher than the older age, and olive oil could increase TGF-β1 levels in the older age guinea pigs. 

 Here are a few questions:

- At first, several references of publications should be added in Discussion. For example, “TGF-β1 can recruit osteoblasts precursors in the area of bone formation and stimulate osteoblasts to differentiate to produce bone matrix” and “During bone formation, TGF-β1 inhibits the recruitment of osteoclast precursor cells and directly inhibitsosteoclast activity in bone resorption.” And please check full of the manuscript.
- In this paper, the number of guinea pigs used in the current experiments is 12. I want to know how did you calculate the required number size and why?
- In addition, as the animals in Olive oil treatment groups were treated with olive oil, were the animals in the control group treated with mock oil?
- Finally, please add more details about methods including Isolation of the GCF and Orthodontic installation in order for peers to reproduce your research.

Is the work clearly and accurately presented and does it cite the current literature?

Partly

If applicable, is the statistical analysis and its interpretation appropriate?

I cannot comment. A qualified statistician is required.

Are all the source data underlying the results available to ensure full reproducibility?

Yes

Is the study design appropriate and is the work technically sound?

Partly

Are the conclusions drawn adequately supported by the results?

Yes

Are sufficient details of methods and analysis provided to allow replication by others?

Partly

Reviewer Expertise:

Mechanisms of orthodontic tooth movement

We confirm that we have read this submission and believe that we have an appropriate level of expertise to confirm that it is of an acceptable scientific standard, however we have significant reservations, as outlined above.

# Response

**Published:** 2020-05-02

**Authors:** Sri Suparwitri

**Affiliations:** Dr., Indonesia

Dear Dr **Mingyuan Du** and Dr **Hong He**

                                                                                         

 Thank you for your kind assistance in reviewing our manuscript and providing us with valuable advice for the manuscript and our future work. We have been working throughout the review. The following are our consolidated comments related to the review and some changes we have done based on the reviewer comments.

- At first, several references of publications should be added in Discussion. For example, “TGF-β1 can recruit osteoblasts precursors in the area of bone formation and stimulate osteoblasts to differentiate to produce bone matrix” and “During bone formation, TGF-β1 inhibits the recruitment of osteoclast precursor cells and directly inhibitsosteoclast activity in bone resorption.” And please check full of the manuscript.

We have edited the discussion section and added other references, Thank you for the punctiliously review. We have added this information in the present manuscript

- In this paper, the number of guinea pigs used in the current experiments is 12. I want to know how did you calculate the required number size and why?

We count it based on the Lemeshow's formula

 (t-1)(n-1)≥ 15

 (7-1)(n-1) ≥15

 n ≥ 3,5

 t represent a total group we determined on this study and n represent the sample. From those formula we have got 3.5 and base on animal restriction we decided to round numbered down into three samples for each group

- In addition, as the animals in Olive oil treatment groups were treated with olive oil, were the animals in the control group treated with mock oil?

Thank you for the punctiliously review. We have added this information in the present manuscript

- Finally, please add more details about methods including Isolation of the GCF and Orthodontic installation in order for peers to reproduce your research.

We have edited the methods section and added this information in the present manuscript.

 We thank you again for the valuable insight.

# Peer review: Reviewer response for version 1

**DOI:** 10.5256/f1000research.23310.r57888

**Published:** 2020-01-13

**Authors:** Ervina Sofyanti, Denny Satria

**Affiliations:** 1 Department of Orthodontics, Faculty of Dentistry, Universitas Sumatera Utara , Medan, Indonesia; 2 Department of Pharmaceutical Biology, Faculty of Pharmacy, Universitas Sumatera Utara, Medan, Indonesia

This title of manuscript has described the main question that addressed by this study. It is relevant and interesting topic in related to relapse of orthodontic treatment. It is necessary to have a scientific background that can show the common thread between previous researches and requirement of adequate orthodontic treatment (including relevant references for previous work and time citation) to understand the motivation and context of research by explaining the experimental approaches and reasons. Such as: The reason of using olive oil as sample is not too clear to prove why the authors using olive oil as sample to increase TGF-B expression in crevicular fluid, and the amount of crevicular fluid that is taken should be explained more detail. 

 Even though the text is clear and easy to be read, this manuscript may require more adequate well-written English and alert to citations. Be careful in citation writing and bibliography. Then, I suggest to re-consider few references in introduction and discussion. Then explaining any steps taken to minimise the effects of subjective bias when allocating animals to treatment and describe who was blinded and when assessing results in this study.

 “ Some researchers show that TGF-β can stimulate osteoclast apoptosis in the area. The effect of TGF-β pro apoptosis is mediated by several factors such as osteoblasts and  stromal  cells  under TGF-β1 control ^5^.” I recommend adding other references. Then I also recommend to re-consider the year of references and add some references in discussion.

 Finally, we recommend to revise this manuscript.

Is the work clearly and accurately presented and does it cite the current literature?

Partly

If applicable, is the statistical analysis and its interpretation appropriate?

Yes

Are all the source data underlying the results available to ensure full reproducibility?

Partly

Is the study design appropriate and is the work technically sound?

Yes

Are the conclusions drawn adequately supported by the results?

Partly

Are sufficient details of methods and analysis provided to allow replication by others?

Partly

Reviewer Expertise:

Dentistry especially related to orthodontics

We confirm that we have read this submission and believe that we have an appropriate level of expertise to confirm that it is of an acceptable scientific standard, however we have significant reservations, as outlined above.

# Response

**Published:** 2020-01-21

**Authors:** Sri Suparwitri

**Affiliations:** Dr., Indonesia

Dear Dr. Ervina and Dr. Denny Satria  

Thank you very much for your kind assistance in reviewing our manuscript. According to your minor suggestions, we have revised within the material and method section.  

This title of the manuscript has described the main question addressed by this study. It is a relevant and interesting topic related to the relapse of orthodontic treatment. It is necessary to have a scientific background that can show the common thread between previous researches and the requirement of adequate orthodontic treatment (including relevant references for previous work and time citation) to understand the motivation and context of research by explaining the experimental approaches and reasons. Such as The reason for using olive oil as the sample is not too clear to prove why the authors using olive oil as a sample to increase TGF-B expression in crevicular fluid, and the amount of crevicular fluid that is taken should be explained more detail.

  Thank you for your kind suggestion. We already added some information about it in the present manuscript. “Food ingredients that contain antioxidants can inhibit the oxidation process in the aging process. The oxidation process can increase intracellular reactive oxygen activity so that it can inhibit bone remodeling. Olive oil is a food ingredient that contains a lot of antioxidants and is often consumed by the public ^7^. Olive oil is extracted from fresh olives have been shown to slow the aging process8. Accord ing to Liu *et al., *olive oil acts effectively as a substitute for estrogen to prevent bone loss9. According to Rodrigues et al., The phenolic compound in virgin olive oil extracts can increase the proliferation and differentiation of osteoblasts. Phenolic compounds (caffeic acid, ferulic acid, coumaric acid, apigenin, and luteolin) in olive oil extracts can increase TGF-β1 and its receptor ^7^.” 

  Even though the text is clear and easy to be read, this manuscript may require more adequate well-written English and alert to citations. Be careful in citation writing and bibliography.    Then, I suggest reconsidering a  few references in the  introduction and discussion. Then explaining any steps taken to minimise the effects of subjective bias when allocating animals to treatment and describe who was blinded and when assessing results in this study. We already added some information about it: “Sample groups were selected using simple random number sampling. Each animal was assigned a unique number, which was picked out of a box by a blindfolded researcher.”

  “ Some researchers show that TGF-β can stimulate osteoclast apoptosis in the area. The effect of TGF-β pro apoptosis is mediated by several factors such as osteoblasts and  stromal  cells  under TGF-β1 control5.” I recommend adding other references. Then I also recommend to re-consider the year of references and add some references in the discussion.   We already added some information about it: “Previous study show that TGF-β1 can stimulate osteoclast apoptosis in the area. The effect of TGF β proapoptosis is mediated by several factors such as osteoblasts and  stromal cells  under  TGF β1 control ^3^.

We also have added and revised our references in the present manuscript

Thank you for the review

Regards
